# Inhibition studies of bacterial α-carbonic anhydrases with phenols

**DOI:** 10.1080/14756366.2022.2038592

**Published:** 2022-02-09

**Authors:** Simone Giovannuzzi, Chad S. Hewitt, Alessio Nocentini, Clemente Capasso, Gabriele Costantino, Daniel P. Flaherty, Claudiu T. Supuran

**Affiliations:** a Neurofarba Department, Pharmaceutical and Nutraceutical Section, University of Florence, Sesto Fiorentino, Italy; b Department of Medicinal Chemistry and Molecular Pharmacology, College of Pharmacy, Purdue University, West Lafayette, IN, USA; c Department of Biology, Agriculture and Food Sciences, CNR, Institute of Biosciences and Bioresources, Napoli, Italy; d Department of Food and Drug, University of Parma, Parco Area delle Scienze, Parma, Italy; e Purdue Institute for Drug Discovery, West Lafayette, IN, USA; f Purdue Institute of Inflammation, Immunology and Infectious Disease, West Lafayette, IN, USA

**Keywords:** Carbonic anhydrase, antibacterials, phenol, *Neisseria gonorrhoeae*, *Vibrio cholerae*

## Abstract

The α-class carbonic anhydrases (CAs, EC 4.2.1.1) from the bacterial pathogens *Neisseria gonorrhoeae* (NgCAα) and *Vibrio cholerae* (VchCAα) were investigated for their inhibition by a panel of phenols and phenolic acids. Mono-, di- and tri-substituted phenols incorporating additional hydroxyl/hydroxymethyl, amino, acetamido, carboxyl, halogeno and carboxyethenyl moieties were included in the study. The best NgCAα inhibitrs were phenol, 3-aminophenol, 4-hydroxy-benzylalcohol, 3-amino-4-chlorophenol and paracetamol, with K_I_ values of 0.6–1.7 µM. The most effective VchCAα inhibitrs were phenol, 3-amino-4-chlorophenol and 4-hydroxy-benzyl-alcohol, with K_I_ values of 0.7–1.2 µM. Small changes in the phenol scaffold led to drastic effects on the bacterial CA inhibitory activity. This class of underinvestigated bacterial CA inhibitors may thus lead to effective compounds for fighting drug resistant bacteria.

## 1. Introduction

**Figure 1.**
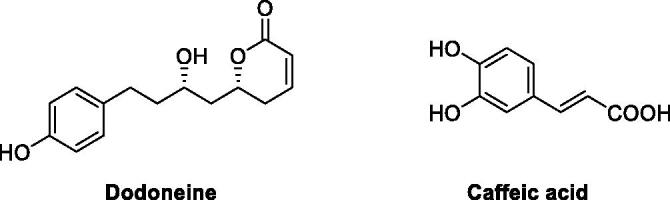
Phenolic derivatives dodoneine isolated from *Agelanthus dodoneifolius,* and caffeic acid, a widespread phenolic acid in many plants.

Phenol (PhOH) was first investigated for its interaction with the metalloenzyme cabonic anhydrase (CA, EC 4.2.1.1) by Koenig’s group in 1980^1^, and few years later Lindskog’s group^2^ demonstrated that this compound is one of the few competitive inhibitors (with CO_2_ as substrate) of these enzymes, more precisely of the human (h) isoform hCA II. However, only in 1994 Christianson’s group^3^ elegantly demonstrated by using X-ray crystallography that phenol shows a new inhibition mechanism against this enzyme: it is anchored to the zinc-coordinated water molecule through hydrogen bonds involving the phenolic OH group, without displacing the zinc-bound water as other classes of inhbitors known at that time^3^. Subsequently, a large number of simple, synthetic and natural product phenols and polyphenols, incorporating various ring systems and possessing a range of substitution patterns were investigated for their inhibitory effects against all mammalian isoforms (CA I–XV) as well as for their interactions with pathogenic bacterial or fungal such enzymes^4–10^. For example dodoneine (Figure 1), a dihydropyranone phenolic compound isolated from the African mistletoe *Agelanthus dodoneifolius*^11^ was demonstrated to possess significant CA inhibitory effects and to induce vasorelaxation through interference with calcium channels blockade and CA inhibition in the vascular smooth muscle cells^11^.

**Figure 2.**
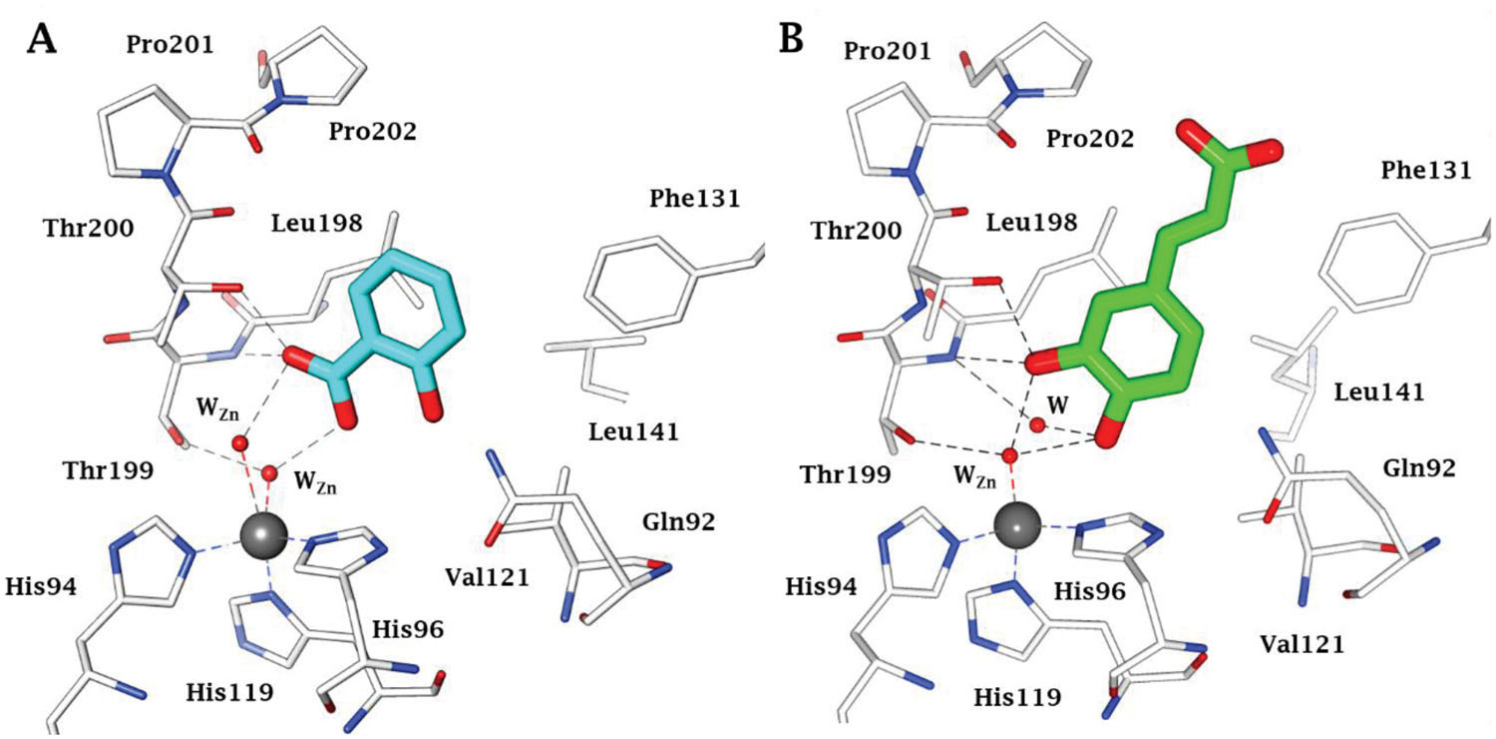
Active site view of hCA II in adduct with A) deacetylated aspirin (PDB 6UX1) and B) caffeic acid (PDB 6YRI). H-bonds are represented as black dashed lines. The active site zinc ion is shown as a grey sphere, and the water molecules as red spheres. Amino acid residues coordinating the metal ion or involved in inhibitor binding are also evidenced.

Such results prompted an intense research in the synthesis of phenolic derivatives incorporating diverse scaffolds (e.g. sugars, steroids, Mannich bases, etc.) ^12^^,^^13^, which have been tested for the inhibition of all hCAs as well as of enzymes belonging to non-α-CA classes, such as β-, γ-, δ-, η-CAs from bacteria, fungi, algae, diatoms and protozoans^14–16^. Recent X-tay crystallographic of aspirin (hydrolyzed to salicylic acid) or caffeic acid bound to hCA II^17^ also allowed a better understanding of the binding mode of such compound to the enzyme (Figure 2) and to rationalise their inhibition mechanism with useful hints for the drug design of novel classes of CA inhibitors (CAIs) ^18^^,^^19^. It may be observed that salicylic acid binds with its carboxylic acid moiety to the zinc-coordinated water molecule through a network of hydrogen bonds, with two water molecules being observed coordinated toi the metal ion^17a^. Caffeic acid anchors with its catechol moiety to the zinc-coordinated water and to the deep water from the CA active site^17b^ – Figure 2.

Such data prompted us to investigate the interactions of a series of simple phenols and some of their derivatives with bacterial CAs which have recently been proposed^20^^,^^21^ as novel drug targets for fighting the emergence of drug resistant bacteria, which no longer respond to clinically used antibiotics^22^. We included in the study NgCAα from *Neisseria gonorrhoeae* and VchCAα from *Vibrio cholerae*.

## 2. Materials and methods

### 2.1. Enzymology and CA activity and inhibition measurements

An Applied Photophysics stopped-flow instrument was used to assay the CA- catalysed CO_2_ hydration activity^23^. Phenol red (0.2 mM) was used as a pH indicator, working at the absorbance maximum of 557 nm, with 10 mM HEPES (pH 7.4) as a buffer, and in the presence of 10 mM NaClO_4_ to maintain constant ionic strength, in order to follow the initial rates of the CA-catalysed CO_2_ hydration reaction for a period of 10–100 s. The CO_2_ concentrations ranged from 1.7 to 17 mM for the determination of the kinetic parameters and inhibition constants. For each inhibitor, at least six traces of the initial 5–10% of the reaction were used to determine the initial velocity. The uncatalyzed rates were determined in the same manner and subtracted from the total observed rates. Stock solutions of inhibitors (10–20 mM) were prepared in distilled-deionized water, and dilutions up to 10 nM were done thereafter with the assay buffer. Inhibitor and enzyme solutions were preincubated together for 15 min prior to the assay, in order to allow for the formation of the E-I complex. The inhibition constants were obtained by non-linear least-squares methods using Prism 3 and the Cheng-Prusoff equation, as reported previously^21^^,^^22^, and represent the mean from at least three different determinations. The NgCAα concentration in the assay system was 7.1 nM whereas the VchCAα was 10.3 nM. The used enzymes were recombinant proteins obtained in-house, as described earlier^22^^,^^24^.

### 2.2. Chemistry

Compounds **1–22**, buffers, acetazolamide **AAZ** and other reagents were of > 99% purity and were commercially available from Sigma-Aldrich (Milan, Italy).

## 3. Results and discussion

Bacterial CAs were investigated in the last decade for their inhibition with the several types of classical CAIs, among which the sulphonamides and their isosteres, the metal complexing anions and more recently also the coumarins^18–22^. However, no detaled inhibition data with many other classes of inhibitors, including phenols, are available so far in the literature for many of these enzymes, among which NgCAα and VchCAα belong^22^^,^^24^. The first of this enzyme was recently validated^21^ as a potential drug target for developing antibiotics able to reverse or at least to alleviate the extensive drug resistance phenomenon that has emerged for this *N. gonorrhoeae*^25^. Although phenols usually act as weaker CAIs compared to other classes such as the sulphonamides or the sulfamates, we decided to investigate here the susceptibility of these enzymes to inhibition by a series of simple phenols and phenolic acids, of types **1–22** (Table 1) investigated earlier for their interaction with the human CA isoforms^4^^,^^6^.

**Table 1.** Inhibition data of human CA isoforms I and II and bacterial NgCAα and VchCAα using **AAZ** as standard drug by a stopped-flow CO_2_ hydrase assay method^23^.

Name	Structure	K_I_ (µM)^a^			
		hCA I	hCA II	NgCAα	VchCAα
**1**	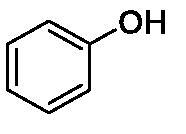	10.2	5.5	0.9	0.8
**2**	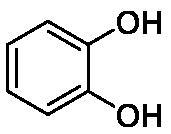	>100	5.5	5.3	20.3
**3**	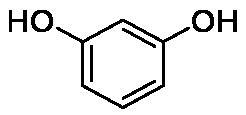	>100	9.4	2.2	8.6
**4**	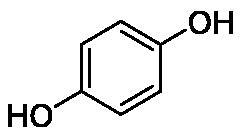	10.7	0.1	4.7	8.4
**5**	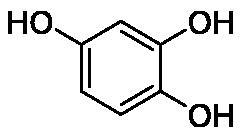	>100	>100	3.9	9.4
**6**	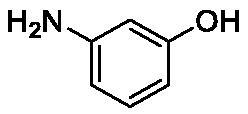	4.9	4.7	1.7	3.9
**7**	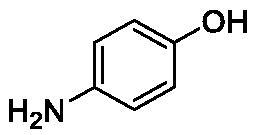	>100	>100	6.0	10.6
**8**	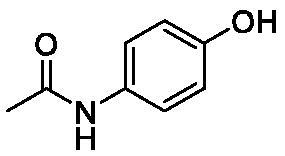	10.0	6.2	1.5	7.0
**9**	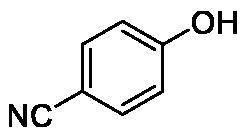	>100	0.1	24.2	17.2
**10**	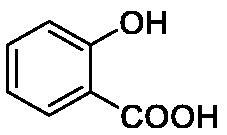	9.9	7.1	43.8	39.1
**11**	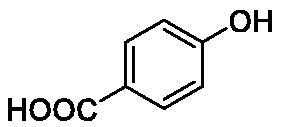	9.8	10.6	8.7	3.5
**12**	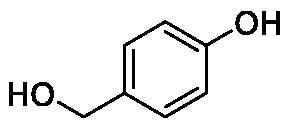	68.9	95.3	0.6	1.2
**13**	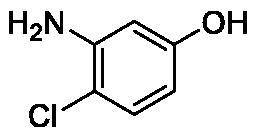	6.3	4.9	0.8	0.7
**14**	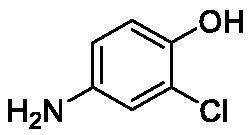	57.8	57.5	71.5	81.6
**15**	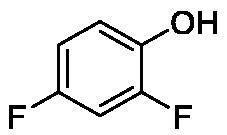	>100	>100	35.9	36.6
**16**	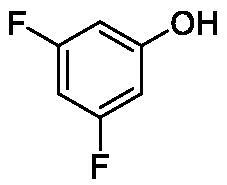	38.8	33.9	3.7	4.9
**17**	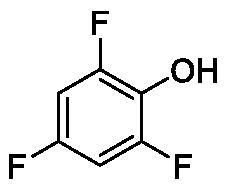	>100	>100	63.1	57.3
**18**	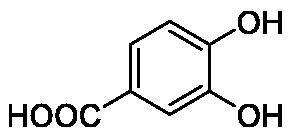	1.1	0.5	9.3	13.8
**19**	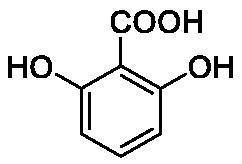	5.7	5.2	33.1	49.6
**20**	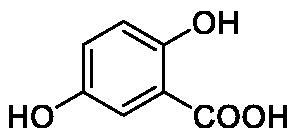	4.2	4.1	16.3	7.3
**21**	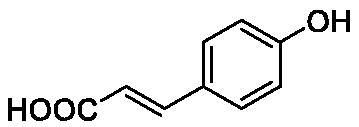	1.1	1.3	10.0	40.5
**22**	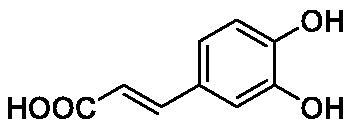	2.4	1.6	76.0	78.1
**AAZ**	–	0.25	0.01	0.075	0.0068

^a^Mean from 3 different assays, by a stopped flow technique (errors were in the range of ± 5–10% of the reported values).

The following structure activity relationship (SAR) can be evidenced from the inhibition data presented in Table 1:

- For NgCAα, the compounds **1-22** investigated here showed inhibitory activity, with K_I_-s in the range of 0.6–76.0 µM. The most effective, submicromolar inhibitors were **1, 6, 12**, **13**, and paracetamol **8**, with K_I_ values of 0.6–1.7 µM. These compounds are the simple phenol, its 4-hydroxymethyl derivative (**12**) as well as 3-amino-phenol or 4-acetamido moiety in derivatives (**6, 8** and **13**). The presence of a second/third H-bond donating moiety such as NH_2_ or OH lead to slight loss of inhibitory activity K_I_ values in the range of 1.7–6.0 µM (Table 1) for derivatives **2-7**. Among this group, moving of the amino group to the *meta*-position in **6** provided the most potent analog at 1.7 µM. The same range of activity is observed also for 4-hydroxy-benzoic acid **11**, the 3,5-difluorophenol **16**, 3,4-dihydroxybenzoic acid **18** and derivative **21** (K_I_ values in the range of 3.7–10.0 µM). However, salicylic acid **10**, 4-cyanophenol **9**, the di-substituted phenols **14** and **15** and caffeic acid **22**, were much less effective inhibitors, with K_I_ values in the range of 24.2–76.0 µM. Thus, relatively small changes in the sscaffold of the phenolic compound lead to drastic changes in the inhibitory activity, with one of the best examples being the pair **21/22**, with the introduction off a second OH moiety in the scaffold of **21** leading to a consistent loss of inhibitory activity. Indeed, caffeic acid **22** is 7.6 times less effective as NgCAα inhibitor compared to **21**.
- VchCAα was inhibited by the investigated compounds with K_I_-s in the range of 0.7–81.6 µM. The most effective inhibitors were phenol **1**, 3-amino-4-chlorophenol **13** and 4-hydroxy-benzyl-alcohol **12** (K_I_-s of 0.7–1.2 µM). Several other phenols, such as derivatives **3-8, 11, 16** and **20** showed effective micromolar inhibition, with K_I_ values in the range of 3.5–10.6 µM, whereas **2, 9, 10, 14, 15, 17-19** and **21, 22** were less effective as VchCAα inhibitors (K_I_ values in the range of 13.8–81.6 µM, Table 21. Again, as for the enzyme discussed above, small structural changes in the molecule of the inhibitor lead to significant sifferences of the inhibitory action (for example compare the three difluorinated phenols **15, 16** and **17**, with one isomer, **16** being an effective inhibitor – K_I_ of 4.9 µM – and the remaining two acting as much weaker inhibitors, with K_I_-s of 36.6 and 57.3 µM, respectively).
- The inhibition pattern of the two bacterial enzymes was generally rather distinct, although some compounds, such as **1, 12, 13** and **16** showed effective inhibition for both of them. However, in most cases, the NgCAα was more susceptible to phenol containing analogs than VchCAα (compare **2, 8, 19** or **21** for their K_I_s). These data points suggest that presumably it may be possible to obtain both phenols that show a wide action against various bacterial CAs, but also selective bacterial CA inhibitors belonging to the phenol and phenolic acid classes of derivatives.
- There were relevant differences of inhibitory activity of the investigated phenols towards the human over the bacterial enzymes (Table 1). Few of the investigated derivatives (e.g. **18, 21, 22**) showed effective hCA I inhibitory activity while moderately inhibiting the bacterial enzymes. Most derivatives were on the other hand poor or ineffective hCA I inhibitors (e.g. **2, 3, 5, 9, 12, 14, 15-17**). hCA II was effectively inhibited by **4, 9, 18** and **20-22**, with few compounds being low activity or inactive as inhibitors (**5, 7, 12, 14-17**) and most of them being moderale, micromolar inhibitors. However, even on this small panel of tested phenols, some compounds showed selectivity form inhibiting the bacterial over the human isoforms, such as for example **12**, and to a lower extent **13**. In fact 4-hydroxy-benzylalcohol has a selectivity index for inhibiting NgCAα over hCA I of 114.8 and over hCA II of 158.8. For VchCAα, the selectivity ratios are of 57.4 (bacterial enzyme over hCA I) and of 79.4 (bacterial enzyme over hCA II). It should be mentioned that benzylalcohol, a compound structurally similar to **12**, was crystallised in adduct with hCA II, and it binds to the enzyme in a similar manner to phenol, by anchoring with the OH group to the zinc-coordinated water molecule^26^.

## 4. Conclusions

We report a study of bacterial CA inhibition with simple phenols and few phenolic acids. Two α-class enzymes from bacterial pathogens which developed drug resistance to classical antibiotics, i.e. NgCAα from *Neisseria gonorrhoeae* and VchCAα from *Vibrio cholerae* were included in the study. The panel of 22 phenols and phenolic acids inhibited both enzymes with K_I_ values in the range of 0.6–76.0 µM for NgCAα, and of 0.6–76.0 µM for VchCAα. The best NgCAα inhibitors were phenol, 3-aminophenol, 4-hydroxy-benzylalcohol, 3-amino-4-chlorophenol and paracetamol, with K_I_s of 0.6–1.7 µM. The most effective VchCAα inhibitors were phenol, 3-amino-4-chlorophenol and 4-hydroxy-benzyl-alcohol, with K_I_-s of 0.7–1.2 µM. Small changes in the phenol scaffold led to drastic effects on the bacterial CA inhibitory activity. This class of underinvestigated bacterial CA inhibitors may thus lead to effective compounds for fighting drug resistant bacteria.

## References

[1] Jacob GS, Brown RD, 3rd, Koenig SH. Interaction of bovine carbonic anhydrase with (neutral) aniline, phenol, and methanol. Biochemistry 1980;19:3754–65. 6773552 10.1021/bi00557a017

[2] Simonsson I, Jonsson BH, Lindskog S. Phenol, a competitive inhibitor of CO_2_ hydration catalyzed by carbonic anhydrase. Biochem Biophys Res Commun 1982;108:1406–12. 6817752 10.1016/s0006-291x(82)80063-6

[3] Nair SK, Ludwig PA, Christianson DW. Two-Site Binding of Phenol in the Active Site of Human Carbonic Anhydrase II: Structural Implications for Substrate Association. J Am Chem Soc 1994;116:3659–60.

[4] a) Innocenti A, Vullo D, Scozzafava A, Supuran CT. Carbonic anhydrase inhibitors: interactions of phenols with the 12 catalytically active mammalian isoforms (CA I-XIV). Bioorg Med Chem Lett 2008;18:1583–7. 18242985 10.1016/j.bmcl.2008.01.077

[5] a) Davis RA, Innocenti A, Poulsen SA, Supuran CT. Carbonic anhydrase inhibitors. Identification of selective inhibitors of the human mitochondrial isozymes VA and VB over the cytosolic isozymes I and II from a natural product-based phenolic library. Bioorg Med Chem 2010;18:14–8. 19962903 10.1016/j.bmc.2009.11.021

[6] Innocenti A, Gülçin I, Scozzafava A, Supuran CT. Carbonic anhydrase inhibitors. Antioxidant polyphenols effectively inhibit mammalian isoforms I-XV. Bioorg Med Chem Lett 2010;20:5050–3. 20674354 10.1016/j.bmcl.2010.07.038

[7] Ekinci D, Kurbanoglu NI, Salamci E, et al. Carbonic anhydrase inhibitors: inhibition of human and bovine isoenzymes by benzenesulphonamides, cyclitols and phenolic compounds. J Enzyme Inhib Med Chem 2012;27:845–8. 21999604 10.3109/14756366.2011.621122

[8] a) Carta F, Vullo D, Maresca A, et al. Mono-/dihydroxybenzoic acid esters and phenol pyridinium derivatives as inhibitors of the mammalian carbonic anhydrase isoforms I, II, VII, IX, XII and XIV. Bioorg Med Chem 2013;21:1564–9. 22668600 10.1016/j.bmc.2012.05.019

[9] a) Ekinci D, Karagoz L, Ekinci D, et al. Carbonic anhydrase inhibitors: in vitro inhibition of α isoforms (hCA I, hCA II, bCA III, hCA IV) by flavonoids. J Enzyme Inhib Med Chem 2013;28:283–8. 22168126 10.3109/14756366.2011.643303

[10] a)Karioti A, Ceruso M, Carta F, et al. New natural product carbonic anhydrase inhibitors incorporating phenol moieties. Bioorg Med Chem 2015;23:7219–25. 26498393 10.1016/j.bmc.2015.10.018

[11] a) Carreyre H, Coustard JM, Carré G, et al. Natural product hybrid and its superacid synthesized analogues: dodoneine and its derivatives show selective inhibition of carbonic anhydrase isoforms I, III, XIII and XIV. Bioorg Med Chem 2013;21:3790–4. 23685174 10.1016/j.bmc.2013.04.041

[12] a) Riafrecha LE, Vullo D, Ouahrani-Bettache S, et al. Inhibition of β-carbonic anhydrases from Brucella suis with C-cinnamoyl glycosides incorporating the phenol moiety. J Enzyme Inhib Med Chem 2015;30:1017–20. 25676329 10.3109/14756366.2014.986120

[13] a) Nocentini A, Bonardi A, Gratteri P, et al. Steroids interfere with human carbonic anhydrase activity by using alternative binding mechanisms. J Enzyme Inhib Med Chem 2018;33:1453–9. Dec 30221552 10.1080/14756366.2018.1512597 PMC7011995

[14] a) Entezari Heravi Y, Bua S, Nocentini A, et al. Inhibition of *Malassezia globosa* carbonic anhydrase with phenols. Bioorg Med Chem 2017;25:2577–82. 28343756 10.1016/j.bmc.2017.03.026

[15] a) Nocentini A, Osman SM, Del Prete S, et al. Extending the γ-class carbonic anhydrases inhibition profiles with phenolic compounds. Bioorg Chem 2019;93:103336. 31604186 10.1016/j.bioorg.2019.103336

[16] Grande R, Carradori S, Puca V, et al. Selective inhibition of helicobacter pylori carbonic anhydrases by carvacrol and thymol could impair biofilm production and the release of outer membrane vesicles. Int J Mol Sci 2021;22:11583. 34769015 10.3390/ijms222111583 PMC8584244

[17] a) Andring J, Combs J, McKenna R. Aspirin: a suicide inhibitor of carbonic anhydrase II. Biomolecules 2020;10:527. 10.3390/biom10040527 PMC7226357 32244293

[18] a) Nocentini A, Angeli A, Carta F, et al. Reconsidering anion inhibitors in the general context of drug design studies of modulators of activity of the classical enzyme carbonic anhydrase. J Enzyme Inhib Med Chem 2021;36:561–80. Dec 33615947 10.1080/14756366.2021.1882453 PMC7901698

[19] a) Supuran CT. Structure-based drug discovery of carbonic anhydrase inhibitors. J Enzyme Inhib Med Chem 2012;27:759–72. 22468747 10.3109/14756366.2012.672983

[20] a) Supuran CT. Bacterial carbonic anhydrases as drug targets: toward novel antibiotics? Front Pharmacol 2011;2:34. 21779249 10.3389/fphar.2011.00034 PMC3132667

[21] a) Supuran CT, Capasso C. Biomedical applications of prokaryotic carbonic anhydrases. Expert Opin Ther Pat 2018;28:745–54. 29973089 10.1080/13543776.2018.1497161

[22] a) Kaur J, Cao X, Abutaleb NS, et al. Optimization of acetazolamide-based scaffold as potent inhibitors of vancomycin-resistant enterococcus. J Med Chem 2020;63:9540–62. 32787141 10.1021/acs.jmedchem.0c00734 PMC8317130

[23] Khalifah RG. The carbon dioxide hydration activity of carbonic anhydrase. I. Stop-flow kinetic studies on the native human isoenzymes B and C. J Biol Chem 1971;246:2561–73. 4994926

[24] a) Del Prete S, Isik S, Vullo D, et al. DNA cloning, characterization, and inhibition studies of an α-carbonic anhydrase from the pathogenic bacterium *Vibrio cholerae*. J Med Chem 2012;55:10742–8. 23181552 10.1021/jm301611m

[25] a) Maduna LD, Peters RPH, Kingsburgh C, et al. Antimicrobial resistance in *Neisseria gonorrhoeae* and *Mycoplasma genitalium* isolates from the private healthcare sector in South Africa: A pilot study. S Afr Med J 2021;111:995–7. 34949296 10.7196/SAMJ.2021.v111i10.15714

[26] De Simone G, Bua S, Supuran CT, Alterio V. Benzyl alcohol inhibits carbonic anhydrases by anchoring to the zinc coordinated water molecule. Biochem Biophys Res Commun 2021;548:217–21. 33647799 10.1016/j.bbrc.2021.02.067

